# Lipid metabolic regulation of neuroinflammation in Alzheimer’s disease

**DOI:** 10.3389/fimmu.2026.1815719

**Published:** 2026-05-12

**Authors:** Tingting Li, Kanglin Guo, Yongxia Ma, Jianjun Zhao, Yanchun Cao, Run Zhang, Xiang Li, Jing Wei, Yufang Ma, Zongxia Zhu, Dongrong Zhao

**Affiliations:** 1 Department of Psychiatry, Lanzhou Second People's Hospital, Lanzhou, Gansu, China; 2 Department of Psychiatry, Lanzhou Third People's Hospital, Lanzhou, Gansu, China; 3 School of Traditional Chinese and Western Medicine, Gansu University of Chinese Medicine, Lanzhou, Gansu, China; 4 Department of Psychiatry, Gansu Provincial Hospital, Lanzhou, Gansu, China

**Keywords:** Alzheimer’s disease, apolipoprotein E (APOE), fatty acids, gut–brain axis, lipid metabolism, metabolic dysregulation, microglia, neuroinflammation

## Abstract

Alzheimer’s disease (AD) is a progressive neurodegenerative disorder characterized by β-amyloid deposition, tau pathology, and sustained neuroinflammation. Increasing evidence indicates that dysregulated lipid metabolism is not merely a metabolic disturbance but a critical modulator of inflammatory responses driving AD pathogenesis. The brain, one of the most lipid-enriched organs, relies on tightly controlled lipid homeostasis to maintain neuronal function and synaptic integrity. Alterations in fatty acid composition, apolipoprotein E (ApoE) isoforms, lipoprotein lipase activity, and lipid-derived signaling mediators profoundly reshape microglial activation states and inflammatory cascades. Obesity, insulin resistance, and gut microbiota dysbiosis further exacerbate systemic and central lipid imbalance, amplifying neuroinflammatory signaling through cytokine networks and blood–brain barrier disruption. Notably, polyunsaturated fatty acids and lipid mediators exert dual immunomodulatory effects, influencing β-amyloid aggregation, oxidative stress, and microglial polarization. This review synthesizes recent advances in understanding how lipid metabolism modulates neuroinflammation and microglia–neuron crosstalk in AD, highlighting emerging therapeutic strategies targeting lipid–inflammation axes as promising avenues for disease modification.

## 1 Introduction

Alzheimer’s disease (AD) represents a relentless, irreversible neurodegenerative condition that primarily targets the elderly population. Clinically, this disorder manifests through a gradual erosion of memory capabilities and cognitive acuity (1, 2). The hallmark neuropathological features of AD include extracellular deposition of β-amyloid (Aβ) plaques and intraneuronal accumulation of hyperphosphorylated tau as neurofibrillary tangles. These pathological alterations drive neuronal injury and loss, ultimately leading to cerebral atrophy and functional deterioration (3). Although the etiology of AD remains incompletely understood, multiple factors, including aging, genetic susceptibility, inflammation, and environmental exposures, have been implicated in disease onset and progression (4).

Lipids, as major energy reservoirs and metabolic substrates, also serve as essential regulators of signaling pathways. The brain is exceptionally lipid-rich; among all human tissues, its lipid content is second only to adipose tissue, accounting for approximately 50% of its dry weight (5). Lipids are therefore indispensable for maintaining normal brain function and overall health (6). Notably, inflammatory responses are tightly coupled to lipid metabolism during the initiation and maintenance of pain, in which lipid species such as fatty acids and lipid-derived signaling mediators play critical roles (7, 8). Disruption of lipid metabolic homeostasis has been linked to a broad spectrum of neurological disorders, including neurodegenerative diseases such as AD (9, 10). Accordingly, delineating lipid metabolic alterations and their functional consequences is crucial for elucidating the mechanisms underlying neurodegeneration. In this review, we summarize and discuss recent advances in understanding how lipids modulate inflammatory processes to influence AD pathogenesis.

## 2 Neuroinflammation in AD

Neuroinflammation refers to inflammatory processes occurring within the central nervous system (CNS), primarily driven by pro-inflammatory mediators released from activated endothelial cells and glial cells (11, 12). These mediators include cytokines, prostaglandins, reactive oxygen species (ROS), and reactive nitrogen species (RNS) (13, 14). Neuroinflammatory responses can induce cerebral edema, tissue injury, and neuronal dysfunction, and are increasingly recognized as critical contributors to cognitive impairment and neurodegenerative disorders (15, 16). The CNS is highly sensitive to inflammatory stimuli (11). A deeper understanding of the mechanisms underlying neuroinflammation and its association with AD may facilitate the development of innovative preventive and therapeutic strategies. Multiple cell types, including microglia, astrocytes, oligodendrocytes, and endothelial cells, actively participate in the neuroinflammatory cascade. A broad spectrum of cytokines, chemokines, and nitric oxide serve as key mediators within this complex inflammatory network (17, 18). NF-κB, the NLRP3 inflammasome, and JAK/STAT signaling are key pathways driving neuroinflammation in AD (19, 20). These pathways can be triggered by Aβ aggregates, tau-associated stress, mitochondrial dysfunction, ROS accumulation, lysosomal damage after Aβ uptake, and inflammatory cytokine stimulation (21, 22). Aβ recognition by pattern-recognition receptors on microglia and astrocytes activates NF-κB, which induces the transcription of pro-inflammatory cytokines and also primes NLRP3 inflammasome activation by increasing NLRP3 and pro-IL-1β expression (23). Transforming growth factor-β (TGF-β), a pivotal immunomodulatory cytokine, exhibits dual roles in AD pathophysiology. On one hand, TGF-β has been implicated in promoting the formation of amyloid deposits within cerebral vasculature; on the other hand, it enhances amyloid plaque phagocytosis while simultaneously suppressing microglial proliferation (24, 25). Moreover, TGF-β is associated with neuronal fiber entanglement and may influence cytoskeletal stability (26). Neuroinflammation also disrupts synaptic plasticity and synaptic pruning, processes essential for neuronal network homeostasis, thereby contributing to neuronal dysfunction and cognitive decline in AD (27).

## 3 Regulation of neuroinflammation by lipid metabolism

### 3.1 Obesity and insulin resistance as modulators of neuroinflammation

**Figure 1:**
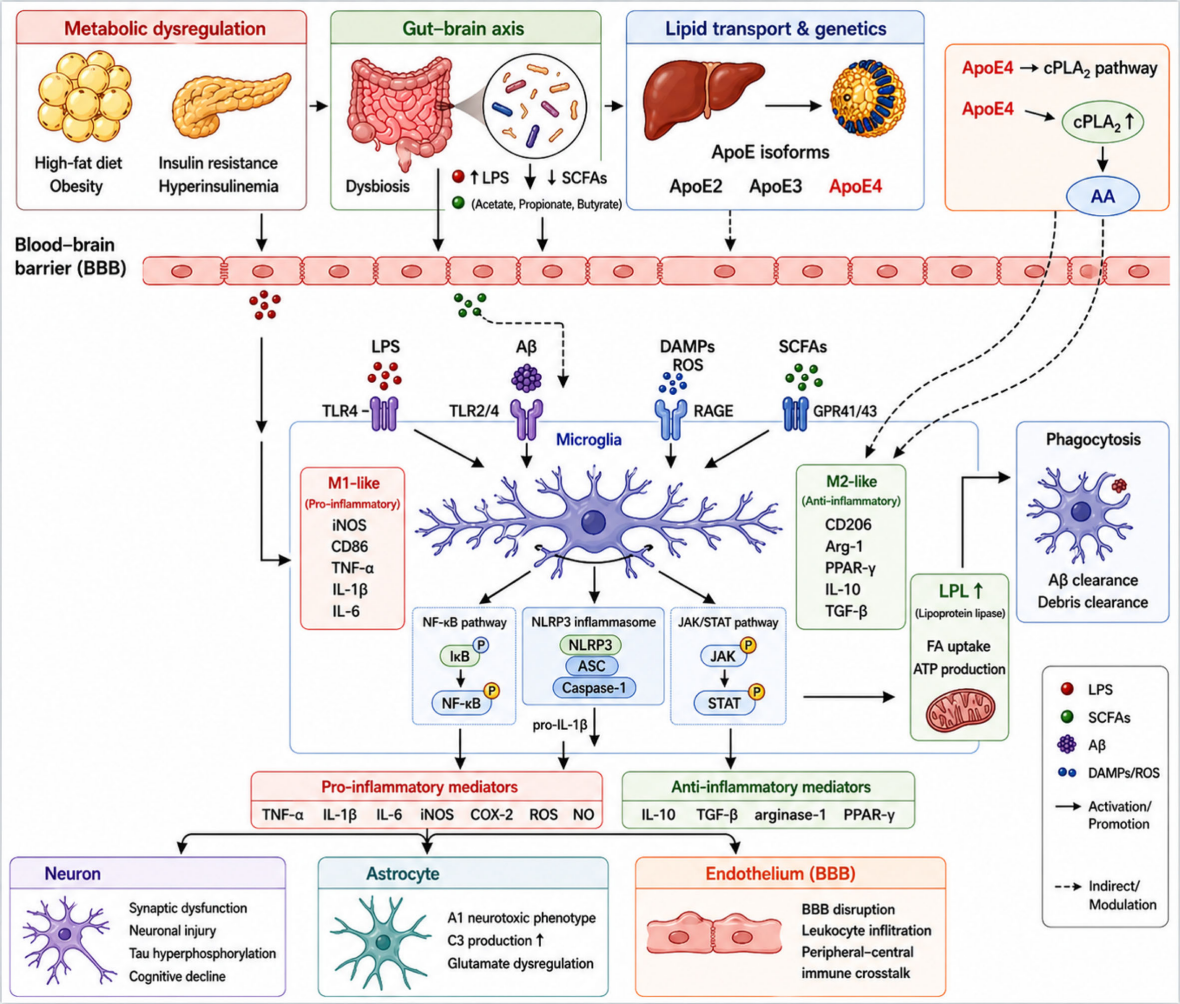
Lipid metabolic regulation of neuroinflammation in Alzheimer’s disease.

Chronic consumption of a high-fat diet disrupts systemic lipid homeostasis and promotes obesity. Increasing evidence recognizes obesity as a state of chronic low-grade inflammation in which adipocytes acquire immune-like properties and secrete a broad spectrum of pro-inflammatory mediators, including IL-6, IL-1β, TNF-α, and various chemokines (28, 29). Neurodegenerative disorders exhibit complex and bidirectional interactions with metabolic dysfunction (30, 31). Obesity may exacerbate central neuroinflammation and contribute to the neuropathological mechanisms underlying AD. Upon activation, microglia and astrocytes generate excessive ROS and RNS. Concurrently, activated glial cells upregulate pro-inflammatory cytokines such as IL-1β and TNF-α and display dysregulated phagocytic activity, ultimately leading to neuronal injury and cell death (32, 33). Insulin resistance, a hallmark of type 2 diabetes mellitus, is increasingly implicated in AD progression. Peripheral insulin resistance results in compensatory hyperinsulinemia, and elevated circulating insulin levels can cross the blood–brain barrier (BBB) (34, 35). In the CNS, hyperinsulinemia promotes the expression of inflammatory cytokines including IL-1β, IL-6, and TNF-α. Notably, inflammation and Aβ deposition reinforce each other, forming a self-perpetuating pathogenic loop that culminates in irreversible neuronal damage (36) (**Figure 1**).

### 3.2 Regulation of neuroinflammation by the gut–brain axis

The gut–brain axis refers to the bidirectional communication network linking the central nervous system and the gastrointestinal tract (37). This complex signaling system operates through coordinated interactions among neural, endocrine, and immune pathways. Inflammatory responses and AD pathogenesis are closely associated with alterations in gut microbial composition (38). Notably, a balanced and diverse intestinal microbiota has been shown to reduce Aβ deposition and delay disease progression (39). Dysbiosis, characterized by compositional and functional shifts in the gut microbiota, increases intestinal permeability and promotes systemic inflammation. This disruption compromises BBB integrity, thereby amplifying neuroinflammatory responses and accelerating AD pathology (40, 41). Microbial metabolites represent key mediators linking gut dysbiosis to central immune activation. For instance, increased systemic levels of lipopolysaccharide (LPS) can activate microglia through TLR4-dependent signaling, leading to downstream activation of NF-κB and MAPK pathways, which promote the production of pro-inflammatory cytokines such as IL-1β, IL-6, and TNF-α (42, 43). In contrast, short-chain fatty acids (SCFAs), including acetate, propionate, and butyrate, exert immunomodulatory effects by engaging G protein-coupled receptors GPR41 and GPR43, thereby regulating microglial activation and inflammatory state (44, 45).

Furthermore, pro-inflammatory microbial metabolites and endotoxins may translocate into the systemic circulation and potentially access the brain, where they impair neuronal function and exacerbate neuroinflammation (46, 47). Alterations in the integrated network comprising the gut microbiota, mucosal immune system, and enteric nervous system may represent a convergent pathway driving neurodegenerative disease onset (48). Beyond immune signaling, the gut microbiota influences AD pathophysiology by modulating endocrine pathways. Microbial communities regulate the secretion of endogenous hormones and neuroactive peptides, including peptide YY (PYY), glucagon-like peptide-1 (GLP-1), ghrelin, leptin, serotonin (5-HT), and cortisol. Dysbiosis alters microbial metabolites—particularly short-chain fatty acids—thereby perturbing the hypothalamic–pituitary–adrenal (HPA) axis and systemic hormonal release. Importantly, SCFAs can cross the BBB and directly affect neuronal function through the gut–brain axis (49). Taken together, the interplay among gut microbiota, the gut–brain axis, and neuroinflammation is intricate and multifaceted. Further elucidation of the microbiome’s mechanistic role in AD initiation and progression may provide novel diagnostic biomarkers and therapeutic strategies.

### 3.3 Role of fatty acids in inflammation

Fatty acids constitute fundamental components of lipids and are broadly classified into saturated fatty acids, trans fatty acids, monounsaturated fatty acids, and polyunsaturated fatty acids (PUFAs) (50, 51). In the CNS, PUFAs are incorporated into membrane phospholipids and influence neuronal survival and apoptosis by modulating membrane fluidity, signal transduction, and gene transcription (52, 53). PUFAs serve as precursors for bioactive lipid mediators that orchestrate inflammatory responses. The dietary ratio of n-3 to n-6 PUFAs critically determines phospholipid composition and downstream lipid mediator profiles (54, 55). These metabolites may exert either pro-inflammatory or anti-inflammatory effects, as well as neuroprotective, antioxidant, and neuromodulatory functions (56). Compared with individuals exhibiting low cortical β-amyloid burden, those with higher amyloid deposition demonstrate elevated plasma arachidonic acid (AA) levels and reduced docosapentaenoic acid (DPA) concentrations (57). Experimental evidence further indicates that increased levels of free fatty acids, particularly cortical free fatty acids, promote the assembly of β-amyloid and tau fibrils *in vitro* (58, 59). Studies investigating six unsaturated fatty acids, including linoleic acid, AA, α-linolenic acid, docosahexaenoic acid (DHA), eicosapentaenoic acid (EPA), and oleic acid, reveal significant associations with neuritic plaque burden and cognitive function. In brain regions vulnerable to AD pathology—such as the mid-frontal and inferior temporal cortices—levels of linoleic acid, α-linolenic acid, and AA are decreased, whereas DHA levels are increased (60). Importantly, these six unsaturated fatty acids directly interact with β-amyloid 40 (Aβ40) and β-amyloid 42 (Aβ42). By interfering with amyloid fibrillogenesis, they exhibit anti-aggregation properties, with oleic acid and DHA demonstrating particularly pronounced inhibitory effects (61).

PUFAs and saturated fatty acids have been demonstrated to directly participate in and modulate microglial inflammatory responses, thereby reshaping innate immune function within the CNS (62, 63). Notably, n-3 PUFAs promote the polarization of microglia toward an anti-inflammatory phenotype and enhance their phagocytic capacity (64, 65). Eicosapentaenoic acid (EPA) and docosahexaenoic acid (DHA) increase the expression of anti-inflammatory markers, including CD206, arginase-1, and peroxisome proliferator-activated receptor-γ (PPAR-γ), while simultaneously suppressing pro-inflammatory gene expression. These regulatory effects attenuate demyelinating pathology in multiple sclerosis models and ameliorate neurodegenerative processes (66).

DHA represents the most abundant PUFA in the human brain (67). Although total fatty acid levels plateau before birth, DHA uniquely continues to accumulate rapidly prior to synaptogenesis (68, 69). With aging, DHA concentrations gradually decline. Physiological aging is associated with global brain atrophy, and reduced DHA levels correlate with hippocampal shrinkage (70, 71). Several studies report decreased brain DHA levels in patients with AD, accompanied by cognitive impairment (72, 73). DHA is primarily obtained from dietary intake or synthesized in the liver (74); however, AD patients exhibit reduced hepatic DHA levels despite elevated levels of short-chain n-3 fatty acid precursors (including docosatetraenoic acid), suggesting impaired DHA biosynthesis (75, 76). Conversely, some studies have reported no significant differences in DHA levels in erythrocytes or brain tissue between AD patients and controls (9, 77), underscoring ongoing controversy. Experimental evidence further demonstrates that substitution of oxidized PUFAs with isotope-reinforced (deuterated) PUFAs suppresses lipid peroxidation. Compared with AD model mice fed oxidized PUFAs, those receiving deuterated PUFAs exhibit reduced hippocampal lipid peroxidation products and decreased β-amyloid production (78).

Oleic acid and arachidonic acid (AA) also display complex and context-dependent associations with AD. In cellular and animal models, oleic acid supplementation reduces β-amyloid formation (79, 80). AD patients exhibit elevated activity of neprilysin, an enzyme implicated in β-amyloid metabolism; among unsaturated fatty acids, oleic acid exerts the strongest inhibitory effect on neprilysin activity (81). However, other studies report that oleic acid increases γ-secretase activity in transfected cells, leading to elevated presenilin-1 and β-amyloid production (82). The relationship between oleic acid, AA, and AD therefore remains controversial. Some epidemiological studies suggest that high dietary intake of oleic acid and AA may increase AD risk (83), whereas others report no significant association (84). Collectively, the interplay between fatty acid metabolism and AD pathogenesis is multifactorial and incompletely understood, warranting further mechanistic and longitudinal investigation (**Supplementary Table 1**).

### 3.4 Regulation of neuroinflammation by apolipoprotein E

ApoE is a ~34 kDa glycoprotein widely expressed in peripheral tissues, including hepatocytes, macrophages, adipocytes, and myocytes. Within the CNS, astrocytes represent the principal source of ApoE, although neurons and microglia also contribute to its production (85, 86). The human ApoE gene is polymorphic, with three major allelic variants—ϵ2, ϵ3, and ϵ4—encoding the corresponding isoforms ApoE2, ApoE3, and ApoE4 (87). Among these, ApoE4 constitutes the strongest genetic risk factor for late-onset AD. Approximately 60–80% of AD patients carry at least one ϵ4 allele. The associated risk is dose-dependent: individuals harboring one ϵ4 allele exhibit a 2–3-fold increased risk of AD, whereas homozygous ϵ4 carriers demonstrate a 10–15-fold elevation in disease susceptibility (88). In AD pathogenesis, ApoE is intimately involved in β-amyloid metabolism, tau-mediated neurodegeneration, neuroinflammatory responses, synaptic integrity, lipid transport, and cerebral glucose metabolism (89). The inflammatory impact of ApoE4 is multifaceted and varies across ethnic backgrounds and environmental contexts (90). Recent studies demonstrate that ApoE4 activates the cytosolic phospholipase A2 (cPLA2) signaling pathway. cPLA2 plays a central role in inflammatory signal transduction and is upregulated within AD plaques. Notably, ApoE4 induces greater cPLA2 activation compared with ApoE3 (91), suggesting that targeting this pathway may provide a therapeutic strategy to mitigate ApoE4-associated neuroinflammation. In addition to its central effects, ApoE4 is linked to systemic inflammation. Carriers of the ϵ4 allele frequently exhibit chronic low-grade peripheral inflammation, which may further amplify AD risk through systemic–central immune crosstalk mechanisms (89).

## 4 The role of microglia in neuroinflammation

Microglia serve as the principal innate immune cells of the CNS and play a pivotal role in neuroinflammatory regulation. One of their primary functions is the surveillance and clearance of toxic protein aggregates. Under physiological conditions, microglia are rapidly activated in response to pathogenic stimuli or misfolded proteins (92, 93). Through macropinocytosis, phagocytosis, and receptor-mediated endocytosis, they internalize pathogenic substrates. Concurrently, microglial activation induces the expression of chemokine receptors, interferon-related genes, and other immune-modulatory factors, contributing to a transient protective inflammatory response (94, 95). Following resolution of the stimulus, microglial activation typically subsides. However, aging impairs microglial surveillance and clearance capacity, rendering these cells susceptible to chronic activation. Aged microglia frequently adopt a pro-inflammatory phenotype characterized by sustained production of inflammatory cytokines, a process implicated in the pathogenesis of neurodegenerative disorders (96, 97). Microglia operate within complex regulatory networks that evolve in parallel with CNS development, maturation, and aging. They are essential for synaptic pruning during development, regulation of neuronal apoptosis, maintenance of synaptic plasticity, and immune surveillance (98). Dysregulated synaptic pruning has been associated with autism spectrum disorders, while impaired immune surveillance and chronic microglial activation are closely linked to neurodegenerative diseases (99).

### 4.1 Microglial phenotypic transformation

Microglia exhibit remarkable phenotypic plasticity, engaging diverse activation pathways that confer complex and context-dependent functions during AD progression (100, 101). In response to pathological stimuli, microglia undergo morphological transformation from a highly ramified, surveillant state to an amoeboid, activated phenotype (102, 103). In the aging brain, microglial branching complexity declines, leading to reduced immune surveillance territories and impaired maintenance of CNS homeostasis (104, 105). Notably, microglial morphology and functional states vary according to spatial localization and temporal stage of disease. Plaque-associated microglia display pronounced morphological remodeling and electrophysiological alterations, whereas microglia located distal to plaques exhibit only modest changes over time (106). These differences likely reflect variations in pathological intensity and duration, as well as stimulus-specific responses to Aβ or tau aggregates (107).

Of particular interest, dystrophic or “dark microglia,” often observed under conditions of metabolic stress, emerge prior to overt tau aggregation (108, 109). Soluble hyperphosphorylated tau can drive microglial phenotypic conversion, impair immune surveillance, and promote AD progression through facilitation of neurofibrillary tangle formation (110, 111). Collectively, morphological, proteomic, and behavioral alterations in microglia correlate closely with disease progression in AD (112, 113). Furthermore, canonical immunological markers shared with macrophages, such as HLA-DR and CD68, lack sufficient specificity to distinguish pro- and anti-inflammatory microglial phenotypes (114). Despite these limitations, the M1/M2 framework remains widely used to illustrate the concept that microglia may exert either protective (M2-like) or detrimental (M1-like) effects depending on pathological context. However, contemporary transcriptomic and single-cell analyses increasingly support a spectrum-based model of microglial activation rather than a rigid binary classification (115, 116).

Lipoprotein lipase (LPL) is a key enzyme involved in triglyceride transport, delivery, and utilization. Dietary patterns rich in carbohydrates or fats have been shown to increase LPL expression in microglia, whereas selective downregulation of microglial LPL reduces lipid uptake and metabolic utilization efficiency. Such alterations lead to mitochondrial morphological abnormalities, insufficient ATP production, and impaired phagocytic capacity (117, 118). In the context of AD, upregulation of microglial LPL promotes fatty acid metabolism and supports bioenergetic homeostasis. Enhanced LPL expression attenuates microglial inflammatory responses and strengthens their capacity to phagocytose β-amyloid, thereby mitigating neuroinflammation and slowing disease progression (118, 119).

### 4.2 Crosstalk between microglia and neurons

Emerging evidence indicates that microglia, astrocytes, and neurons engage in tightly coordinated interactions that collectively drive neuroinflammatory pathology. In AD, Aβ activates the NF-κB signaling pathway in astrocytes, resulting in increased release of complement component C3. C3 subsequently acts on C3a receptors (C3aR) expressed on neurons and microglia, leading to neuronal dysfunction and further microglial activation (120, 121). Activated microglia, in turn, secrete IL-1α, complement component C1q, and tumor necrosis factor (TNF), which induce the formation of neurotoxic astrocytes (122, 123). Within an inflammatory milieu, bidirectional signaling between microglia and astrocytes establishes a positive feedback loop that amplifies inflammatory cascades and disrupts homeostatic regulation (124, 125). Importantly, neuron–glia communication is also profoundly altered in AD. The CD200R signaling axis and the fractalkine pathway, comprising CX3CL1 and its receptor CX3CR1, are critical regulators of neuron–microglia crosstalk and are essential for maintaining microglial homeostasis (126). Under physiological conditions, neuronal CD200 and CX3CL1 provide inhibitory signals that restrain microglial activation. However, in the AD brain, expression of CD200, CD200R, and CX3CR1 is reduced, weakening neuron-derived inhibitory signaling and exacerbating microglial activation and neuroinflammatory responses (127, 128).

## 5 Conclusion

Lipid metabolism and neuroinflammation are dynamically interconnected processes that collectively shape the pathophysiological landscape of Alzheimer’s disease. Mounting evidence supports the concept that disrupted lipid homeostasis is not merely a secondary consequence of neurodegeneration but an active driver of inflammatory amplification, β-amyloid accumulation, and neuronal dysfunction. Altered fatty acid profiles, oxidative lipid damage, ApoE isoform–dependent lipid transport, and metabolic comorbidities such as obesity and insulin resistance converge to modulate microglial activation states and inflammatory mediator production. In particular, ApoE4-associated lipid remodeling and cPLA2 pathway activation provide mechanistic insight into how genetic susceptibility intersects with inflammatory signaling networks.

Microglia occupy a central position in this regulatory axis. Their metabolic programming, lipid uptake capacity, and phenotypic plasticity determine whether inflammatory responses remain protective or become chronically deleterious. Emerging evidence from lipidomics, transcriptomics, and microbiome research further underscores the role of gut-derived lipid metabolites and systemic metabolic signals in shaping central immune responses. However, inconsistencies in clinical findings—particularly regarding polyunsaturated fatty acid levels—highlight the complexity and heterogeneity of lipid–inflammation interactions in AD. Future research should integrate multi-omics approaches, longitudinal clinical studies, and mechanistic experimental models to clarify causal relationships. Targeting lipid metabolic pathways, restoring microglial metabolic balance, and modulating lipid-derived inflammatory mediators may represent promising strategies for disease modification. A deeper understanding of lipid–immune crosstalk will be essential for developing precision therapies aimed at halting or slowing Alzheimer’s disease progression.
